# A sportomics strategy to analyze the ability of arginine to modulate both ammonia and lymphocyte levels in blood after high-intensity exercise

**DOI:** 10.1186/1550-2783-9-30

**Published:** 2012-06-26

**Authors:** Luis Carlos Gonçalves, Artur Bessa, Ricardo Freitas-Dias, Rafael Luzes, João Pedro Saar Werneck-de-Castro, Adriana Bassini, Luiz-Claudio Cameron

**Affiliations:** 1 Laboratory of Biochemistry of Proteins, Federal University of State of Rio de Janeiro, Av. Pasteur 296, CEP: 22290-240, Rio de Janeiro, Brazil; 2 Institute of Genetics and Biology, Federal University of Uberlândia, Av. João Naves de Ávila 2121, CEP: 38408-100, Uberlândia, Brazil; 3 Laboratory of Biology of Exercise, Federal University of Rio de Janeiro, Av. Carlos Chagas Filho 540, CEP: 21941-599, Rio de Janeiro, Brazil; 4 University Castelo Branco, Av. Salvador Allende 6.700, CEP: 22780-160, Rio de Janeiro, Brazil

**Keywords:** White blood cells, Granulocytes, Amino acids, Immune response, Short-duration exercise

## Abstract

**Background:**

Exercise is an excellent tool to study the interactions between metabolic stress and the immune system. Specifically, high-intensity exercises both produce transient hyperammonemia and influence the distribution of white blood cells. Carbohydrates and glutamine and arginine supplementation were previously shown to effectively modulate ammonia levels during exercise. In this study, we used a short-duration, high-intensity exercise together with a low carbohydrate diet to induce a hyperammonemia state and better understand how arginine influences both ammonemia and the distribution of leukocytes in the blood.

**Methods:**

Brazilian Jiu-Jitsu practitioners (men, n = 39) volunteered for this study. The subjects followed a low-carbohydrate diet for four days before the trials and received either arginine supplementation (100 mg·kg^-1^ of body mass·day^-1^) or a placebo. The intergroup statistical significance was calculated by a one-way analysis of variance, followed by Student’s *t*-test. The data correlations were calculated using Pearson’s test.

**Results:**

In the control group, ammonemia increased during matches at almost twice the rate of the arginine group (25 mmol·L^-1^·min^-1^ and 13 μmol·L^-1^·min^-1^, respectively). Exercise induced an increase in leukocytes of approximately 75%. An even greater difference was observed in the lymphocyte count, which increased 2.2-fold in the control group; this increase was partially prevented by arginine supplementation. The shape of the ammonemia curve suggests that arginine helps prevent increases in ammonia levels.

**Conclusions:**

These data indicate that increases in lymphocytes and ammonia are simultaneously reduced by arginine supplementation. We propose that increased serum lymphocytes could be related to changes in ammonemia and ammonia metabolism.

## Background

We previously proposed that exercise can be used as a tool to study the interactions between metabolic stress and the immune system [1,2]. Exercise can be employed as a model of the temporary immunosuppression that occurs after severe physical stress [3,4]. Exercise impacts the immune response, and these effects depend on the intensity, duration and nature of the exercise [5]. Changes in the number of white blood cells (WBC) in circulation were previously described in various exercise experiments, including both high- and low-intensity exercises [6,7]. The immunological effects caused by exercise have been associated with the mechanical release of leukocytes from the vessel walls due to increased blood flow or catecholamine release, which is a mechanism that can be partially explained by cell adhesion signaling [8,9].

Hyperammonemia can be caused by urea cycle enzyme diseases, liver failure and exercise (for a recent review, see Wilkinson et al. [10]). In general, ammonia (which here refers to the sum of NH_3_ and NH_4_^+^) is highly toxic to humans, and hepatocytes maintain the blood concentration of ammonia in the 20–100 μM range. Ammonia can cross the blood–brain barrier and reach levels greater than 800 μmol/L inside the central nervous system (CNS), which can lead to a decrease in cerebral function, neuropsychiatric disorders and death [11]. Ammonia-mediated excitotoxicity has been proposed as a mechanism for spreading damage in the CNS [12].

Ammonia levels change over time, and data obtained from exercises of different intensities have been used to help explain the effects of transient hyperammonemia [6,13]. A rise in ammonemia occurs after different types of exercise, and these changes can be managed by supplementation with amino acids or carbohydrates, which interfere with ammonia metabolism [13,14]. In addition, we recently showed that a mixture of amino acids and ketoacids can interfere with the increase in ammonemia in both human and rat exercise studies [15,16].

Arginine (Arg) has a versatile metabolic role in cell function. It can be used as a precursor not only for protein synthesis but also for the synthesis of nitric oxide, urea, and other amino acids, such as glutamate [17]. Exercise studies show that mammals that receive Arg supplementation have greater concentrations of urea cycle intermediates in the serum, less lactatemia and better ammonia buffering than controls [18,19]. Arg supplementation has also been described as an immune system stimulator, mainly in the production of T cells [20,21].

We used a sportomics approach to understand exercise-induced cellular and metabolic modifications in a field experiment [22,23]. Sportomics is the use of “-omics” sciences together with classical clinical laboratory analyses (e.g., enzymatic determinations, ELISA and western blotting) to understand sport-induced modifications. The suffix “-ome” means that all constituents are considered collectively; therefore, for example, proteomics is the study of all proteins, and metabolomics is the study of all metabolic processes. We treated data in a systemic way and generated a large amount of data in a type of non-target analysis using a top-down approach.

Here, we combined a high-intensity exercise with a previously described low-carbohydrate diet [16], which act synergistically to increase ammonemia, to better understand the ability of arginine to modulate both ammonia and leukocyte changes in the blood.

## Methods

### Subjects

World-class Brazilian Jiu-Jitsu competitors (n = 50 males) volunteered to take part in this double-blind, randomized study. Because they had difficulty in following the diet, 11 subjects withdrew from the study. The enrolled athletes had a minimum of three years of Jiu-Jitsu experience. Users of pharmaceutical drugs or nutritional ergogenic aids were excluded from the study. The included athletes had not sustained any injuries in the previous six months.

The subjects were randomly divided into two groups. The arginine-supplemented group (RG, n = 16) ingested 100 mg·kg^-1^ of body mass·day^-1^, and the control group (PG, n = 23) took 100 mg·kg^-1^ of body mass·day^-1^ of lactose with supplement doses as described previously [18,24]. Each athlete received packs of indistinguishable capsules containing the daily doses and used them for four days, including the day of the experiment.

The athletes were briefed about the aim and the protocol of the study. Informed written consent was obtained from all of the subjects, and the experiments were performed in accordance with the guidelines from the ethics committee for human research of the Universidade Federal do Estado do Rio de Janeiro and the requirements for performing research on human subjects (Health National Council, Brazil, 1996).

### Diet

Athletes from both groups followed a low-carbohydrate diet (LCD) as previously described [16]. LCD adherence was verified by diet evaluation before the experiment and ketonuria. The athletes refrained from caffeine, ethanol and smoking for three days before the trials. To decrease the glycogen stores, the experiment was conducted after 12 h of fasting. The last supplement doses were given 90 min before the match.

### Experiment

The participants engaged in a six-minute Brazilian Jiu-Jitsu match in full gear. The matches were performed at similar temperatures and levels of humidity and began with the athletes kneeling to avoid injuries from falling. The subjects were instructed to maintain high mobility and avoid finishing the match. The opponent in the match was not subjected to an LCD and was exchanged for a rested opponent after 3 minutes of elapsed match time to maintain an intensity that was as high as possible in the study subjects. The matches occurred between individuals in the same weight category.

The exercise intensity was evaluated during a pilot experiment, and the athletes displayed a range from 85% to 90% of their maximum heart rate; we also observed that the match promoted a similar kinetic ammonia serum increase for all athletes (data not shown).

### Blood sampling

Blood samples were collected following venipuncture at rest immediately before and ~1, 3, 5, 7 and 10 min after the match. Because the fight took 6 minutes, the data in the figures are shown with times 7, 9, 11, 13 and 16 min after the beginning of the exercise. The pre-match sample was collected just before the exercise began, which occurred 90 min after the last dose of arginine or lactose, and the first post-match sample was collected immediately after the match ended (30–60 sec). Blood was collected in tubes containing K_3_EDTA and refrigerated until the hematological analysis (up to 2 h).

The blood analyses were performed at least in triplicate. Red blood cells and platelets were also measured. Samples for the biochemical assay were collected in tubes with a coagulation enhancer and splitting gel (Vacuette, Greiner Bio-One) and immediately centrifuged (3,000 × g, 10 min). The blood serum was aliquoted and stored in liquid nitrogen for future analyses. The sera were analyzed using clinical kits for the following muscle injury markers and biochemical variables: ammonia, creatine kinase (CK), creatine kinase-MB (CK-MB), aspartate aminotransferase (AST), alanine aminotransferase (ALT), γ-glutamyltransferase (γGT), lactate dehydrogenase (LDH), alkaline phosphatase (ALP), glucose, urea, creatinine, urate, total protein, albumin, bilirubin, globulins, and serum hemoglobin. No changes in plasma volume were detected during the experiment.

### Calculations and statistics

The area under the curve (AUC) for the blood ammonia data for each individual in each treatment was determined using the equation AUC = {Ai(Ti + 1 - Ti) + (1/2)(Ai + 1 - Ai)(Ti + 1 - Ti)}, where A denotes ammonia concentration (μmol/L) and T denotes time (min). The blood ammonia accumulation rate during the match was calculated by the difference between the ammonia concentrations before and approximately 1 minute after exercise divided by 6 minutes.

The data are shown as the mean and standard error. The data were normalized to the pre-exercise values. The intergroup statistical significance was calculated by a one-way analysis of variance (ANOVA), and the intragroup significance was established by Student’s *t*-test. The data correlations were calculated using Pearson’s test. Significant differences were assumed at P < 0.05.

## Results

### Proteins and injury markers

To ensure that the athletes were at similar training levels and had similar liver integrities, we measured the classic muscle and liver injury markers. The athletes of both groups had similar anthropometric values (Table 1). Despite the high levels of classic muscle injury markers, such as CK (EC 2.7.3.2) and LDH (EC 1.1.1.27), the concentrations of these enzymes in the blood did not change after the match. The liver injury markers ALP (EC 3.1.3.1) and γGT (EC 2.3.2.2) also remained stable in both groups. The same stability was observed with the less specific markers AST (EC 2.6.1.1) and ALT (EC 2.6.1.2) (Table 2). The amount of globulins in the blood increased in both groups after exercise, with an 11% increase in the RG and a 15% increase in the PG (Table 2).

**Table 1:** Age and anthropometric measurements in Brazilian Jiu-Jitsu fighters assigned to the PG and RG

	**PG**	**Range**	**RG**	**Range**
Age (years)	25.2 ± 0.4	21-28	26.2 ± 0.6	23-29
Weight (kg)	82.2 ± 1.8	70-103	79.2 ± 3.2	65-120
Height (cm)	177 ± 1.0	170-188	175 ± 1.4	170-190

Values are the mean ± SE and the range. No significant differences were detected (p > 0.05).

**Table 2:** Muscle and liver injury markers measured before (PRE) and after the match (POST)

	**PG**		**RG**	
	**PRE**	**POST**	**PRE**	**POST**
CK (U/L)	737.0 ± 187.2	1051.2 ± 401.9	559.1 ± 128.3	625.1 ± 148.8
CKMB (U/L)	13.6 ± 4.1	36.0 ± 13.5	12.2 ± 2.0	17.6 ± 3.2
LDH (U/L)	390.0 ± 41.9	402.8 ±29.6	354.6 ± 18.4	388.3 ± 17.9
δGT (U/L)	21.7 ± 2.4	21.7 ± 2.7	27.4 ± 4.2	30.2 ± 4.4
ALP (U/L)	80.8 ± 11.8	88.1 ± 12.0	67.6 ± 7.7	74.6 ± 7.4
ALT (U/L)	23.0 ± 3.8	26.2 ± 3.2	30.1 ± 5.2	29.9 ± 5.1
AST (U/L)	52.7 ± 17.9	68.2 ± 21.2	36.0 ± 3.7	45.2 ± 5.8
Albumin (g/L)	43.3 ± 0.2	46.0 ± 0.2	45.9 ± 0.2	50.2 ± 0.2
Globulins (g/L)	32.5 ± 0.1	38.0 ± 0.1	31.1 ± 0.1 #	34.6 ± 0.1 #

PG, placebo group; RG, arginine group. Values are the mean ± SE and the range. No statistically significant inter- or intragroup differences were detected, except for globulins in response to exercise and to supplementation.

### Ammonia and its metabolites

**Figure 1:**
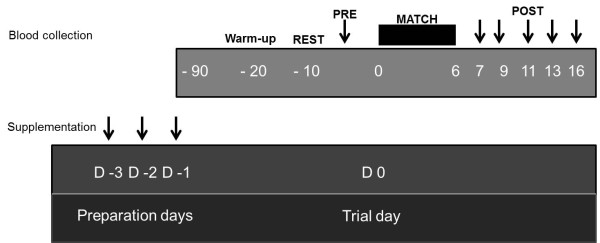
**Experimental design.** Before the experiment, the athletes were subjected to a four-day LCD as described in the Materials and Methods. Blood was collected before the athletes received supplementation (PRE). Warm-up and exercise protocols were performed, followed by six blood collections immediately after exercise (POST; 1, 3, 5, 7 and 10 min).

**Figure 2:**
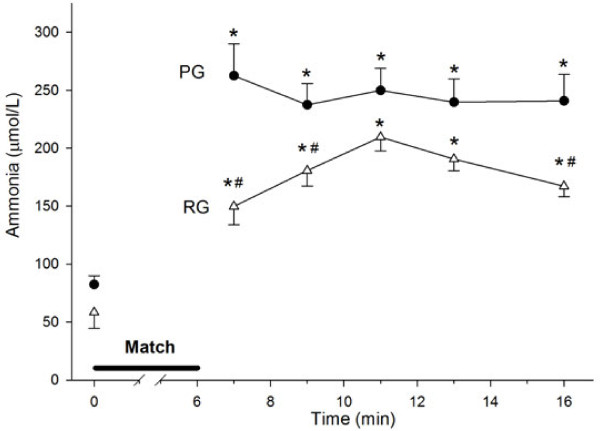
**Blood ammonia concentration increases after a high-intensity exercise in an arginine supplementation-dependent manner.** A six-minute Jiu-Jitsu match was performed after a three-day LCD by athletes who had received either arginine (RG, Δ) or a placebo (PG, ●). Blood was collected before and after exercise and treated as described in the Materials and Methods. Control, n = 23; Arginine, n = 16. (*) denotes that the average ± SE is different from the pre-exercise values; (#) denotes a difference between the two experimental groups. The calculated area under the curve was 3397 μmol/L·min-1 for the placebo group and 2366 μmol/L·min-1 for the arginine group.

To evaluate the consequences of an increase in the blood ammonia concentration induced by high-intensity exercise, we used a Brazilian Jiu-Jitsu match as an exercise stress inducer (Figure 1). In the control group, ammonemia increased during the match at almost twice the rate of the RG (25 μmol·L^-1^·min^-1^ and 13 μmol·L^-1^·min^-1^, respectively). The AUC analysis showed that the RG maintained lower ammonemia (~30%) compared with the controls (Figure 2).

**Figure 3:**
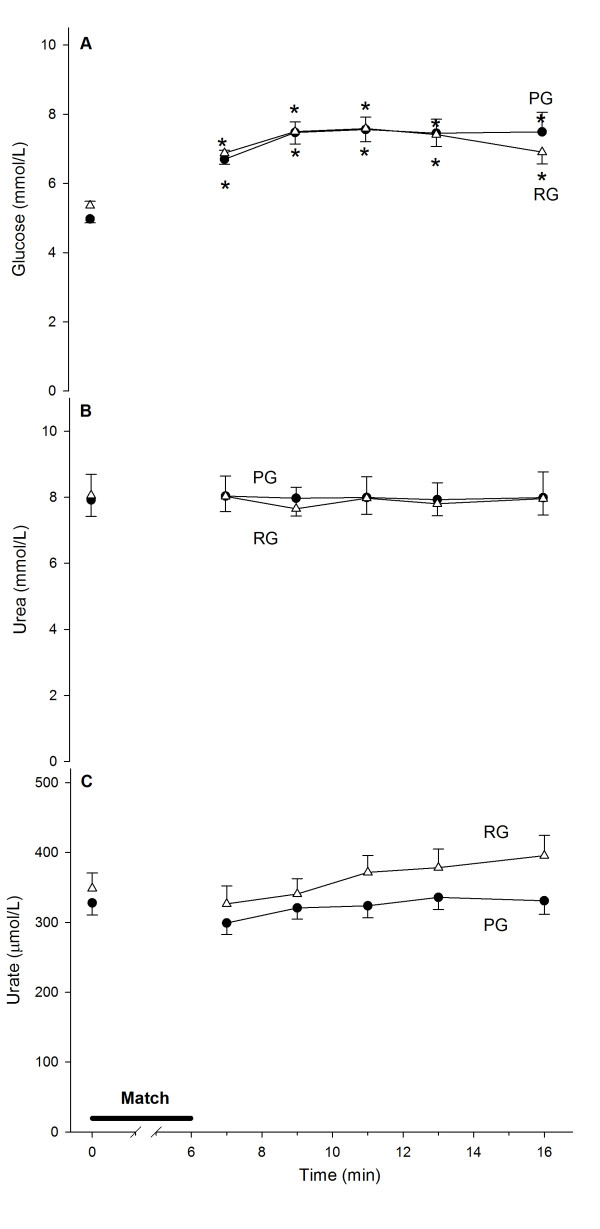
**Glucose increases in response to exercise in a supplementation-independent manner (A). Neither supplementation nor exercise affects urea (B) or urate (C) after intense exercise.** Control, n = 23 (PG, ●); Arginine, n = 16 (RG, Δ). (*) denotes that the average ± SE is different from the pre-exercise values.

We measured the glycemia changes as a control for Arg supplementation. The match led to a 30% increase in glycemia in both groups, and glycemia remained high until the last measurement, which occurred ten minutes after the match (Figure 3A). To evaluate the urea increase due to the higher ammonia production, we measured the urea level in the blood. There were no differences in the blood urea concentration between the groups either before or after exercise (Figure 3B). Ammonia production during high-intensity exercise is mainly due to AMP deamination, which leads to IMP. The final product of IMP metabolism is urate. There were no changes in the blood urate concentration between the groups either before or after the match (Figure 3C). None of the above metabolites showed changes in response to Arg supplementation when we compared the pre- and post-match levels (Figure 3).

### Blood cells

**Figure 4:**
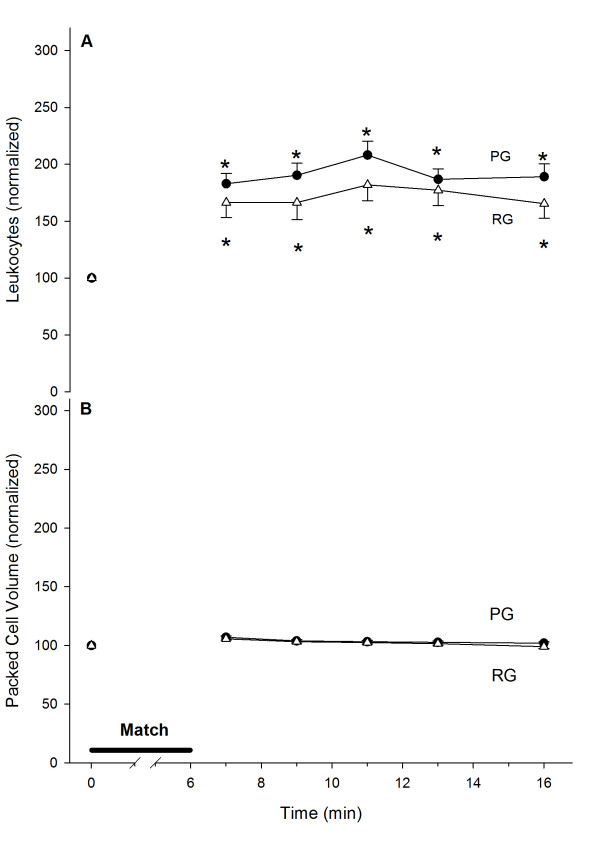
**White blood cell counts increase (A) after intense exercise without changes in packed cell volume (B).** Control, n = 23 (PG, ●); Arginine, n = 16 (RG, Δ). (*) denotes that the average ± SE is different from the pre-exercise values. The absolute pre-exercise WBC counts are 5.9 ± 0.2 cells × 10^9^/L for the PG and 6.4 ± 0.5 cells × 10^9^/L for the RG; the packed cell volumes are 47.5 ± 0.6% for the PG and 46.6 ± 0.6% for the RG.

The six minutes of exercise induced an increase in leukocytes of approximately 75% in both groups. This elevated level did not decrease in the ten minutes following the experiment and was similar between the groups (Figure 4A). To avoid misinterpretations due to volemic variations, we also evaluated the red blood cell counts. The packed cell volume was not altered by exercise (Figure 4B). We did not detect any differences in the red blood cell count, volume or hemoglobin content in response to either exercise or supplementation.

**Figure 5:**
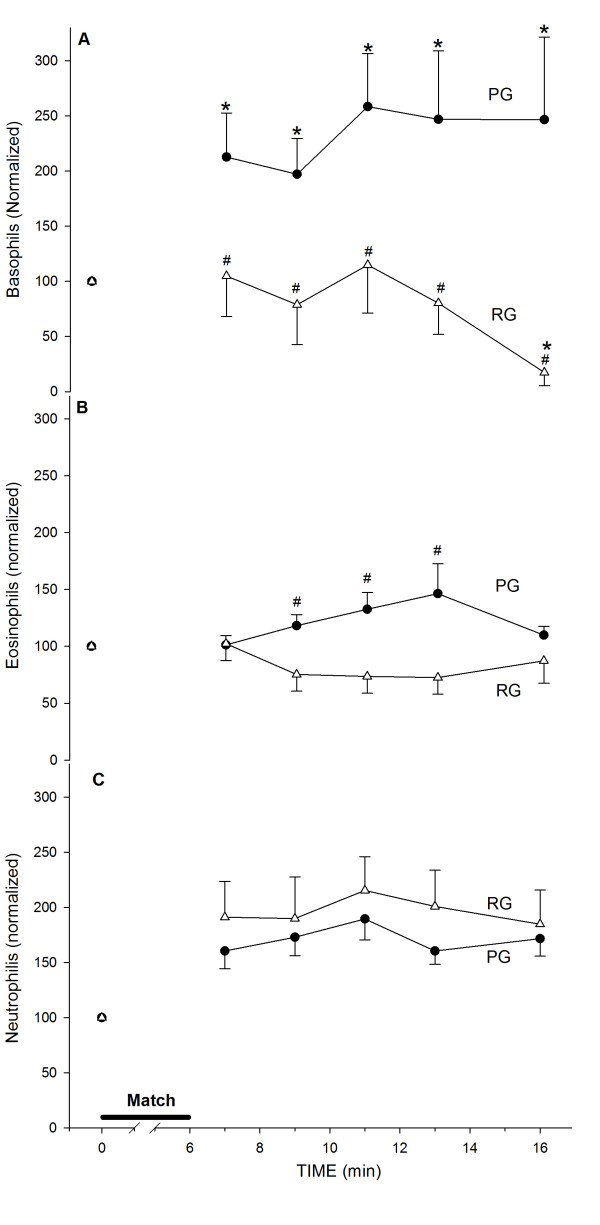
**Granulocyte counts in response to exercise and supplementation. Basophils (A); eosinophils (B); neutrophils (C).** Control, n = 23 (PG, ●); Arginine, n = 16 (RG, Δ). (*) denotes that the average ± SE is different from the pre-exercise values; (#) denotes a difference between the experimental groups. The absolute pre-exercise values for basophils are 2.6 ± 0.4 × 10^7^ cells /L for the PG and 1.9 ± 0.9 × 10^7^ cells /L for the RG; for eosinophils, 1.8 ± 0.3 × 10^8^ cells /L for the PG and 2.0 ± 0.5 × 10^8^ cells /L for the RG; and for neutrophils, 3.1 ± 0.2 × 10^9^ cells /L for the PG and 2.7 ± 0.4 × 10^9^ cells /L for the RG.

**Figure 6:**
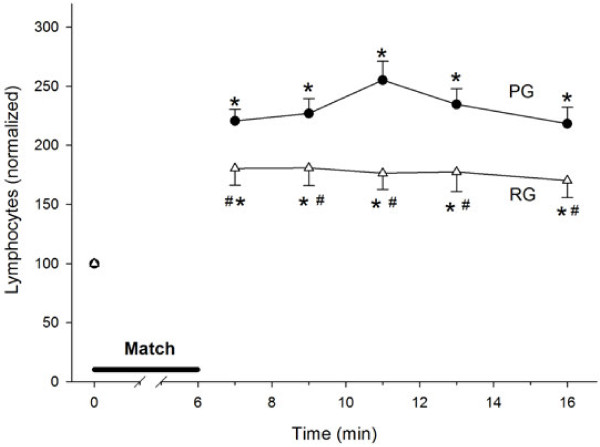
**Exercise induces an increase in lymphocytes in an arginine supplementation-dependent manner.** Control, n = 23 (PG, ●); Arginine, n = 16 (RG, Δ). (*) denotes that the average ± SE is different from the pre-exercise values. The absolute pre-exercise values are shown within the graphs. The absolute pre-exercise values for lymphocytes are 2.2 ± 0.1 × 10^9^ cells /L for the PG and 2.9 ± 0.3 × 10^9^ cells /L for the RG (no statistically significant difference, p = 0.07).

Differential white blood cell analyses showed a distinct response to both exercise and Arg supplementation. The basophil counts rose two-fold in the PG but did not change in the RG (Figure 5A). The eosinophil counts were significantly different between the groups after the end of exercise (Figure 5B). However, neutrophils appeared not to respond significantly in either the PG or RG (Figure 5C). The exercise led to a 2.2-fold increase in the lymphocyte count. This increase was significantly reduced by Arg supplementation (Figure 6A).

**Figure 7:**
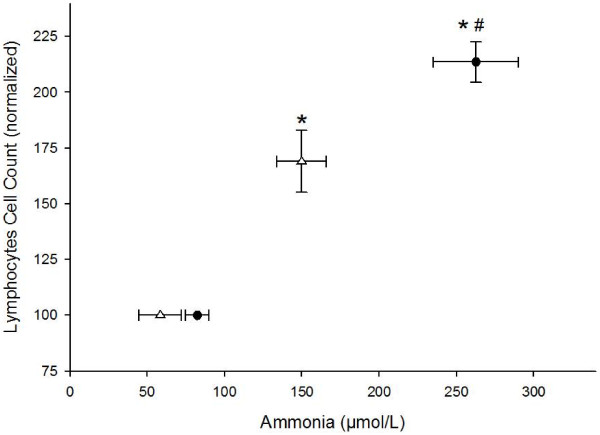
**Ammonemia increase is related to the blood lymphocyte count.** The lymphocyte count is plotted against ammonemia. (*) denotes that the average ± SE is different from the pre-exercise values; (#) denotes a difference between the experimental groups. Pearson correlations indicate that the relationship between the lymphocyte count and ammonemia is indirect. The lymphocyte increases were normalized to pre-fight levels to ensure a better understanding of the results. Control, n = 23 (PG, ●); Arginine, n = 16 (RG, Δ).

To better understand the ammonia–lymphocyte relationship with Arg supplementation during exercise, we plotted the ammonia response to exercise against the lymphocyte count. The exercise-induced increases in ammonia and the lymphocyte count were highly correlated. The lymphocyte count associated with the increase in ammonia was decreased by Arg supplementation (Figure 7).

## Discussion

Ammonia has deleterious effects on many systems, including the CNS, and has been identified as a potential cause of central fatigue. Blood ammonia is normally in the range of 20–100 μM, and concentrations above this range have been correlated with the incidence of encephalopathy, coma and death [10]. During exercise, ammonemia can exceed 350 μM without obvious symptoms [13]. In this study, we used an LCD (to deplete glycogen stores) combined with a Brazilian Jiu-Jitsu session using a sportomics protocol to investigate the increase in blood ammonia and changes in the white blood cell levels following exercise.

The blood ammonia increased four- to six-fold after a six-minute match and reached levels as high as 610 μM in one individual. These values are higher than the published averages, even if we consider other match-based studies [6,25], which confirms that this experimental protocol is a powerful short-term metabolic stress inducer. The velocity of the ammonia increase was partially (50%) retarded by previous Arg intake, and the total ammonia was lower in the RG. In addition, the analysis of individual ammonia clearance suggests a greater velocity in the supplemented group.

An increase in blood ammonia depends on different factors, including glycogen stores, amino acid deamination and glucose availability [26]. We used this knowledge as the rationale for depleting the glycogen stores using an LCD. In our study, blood glucose increased up to 30% in response to exercise and remained at this elevated level until the final measurement ten minutes after the match irrespective of Arg supplementation. This finding rules out an effect of Arg on ammonemia due to Arg supplementation-induced glucose production. The shape of the ammonemia curve suggests that Arg promotes buffering against the ammonemia increase likely by increasing ammonia clearance via the promotion of higher levels of urea cycle intermediates, as previously described [18].

We performed a biochemical pre-evaluation of our subjects to assess the integrity of their liver function. The liver function of the athletes was assessed based on their hepatic metabolic function and hepatocyte integrity, which were measured by the presence of intracellular hepatocyte enzymes in blood. Neither blood urea nor urate production showed any significant differences between the groups before or after exercise. This finding is acceptable because we measured the total production of both metabolites in the blood over a short time period. The long-term supplementation of both glutamine and alanine increases the resting level of blood urea [13]. In this study, we did not find any differences in urea or urate at rest between the groups. Both groups had a similarly increased basal urea level compared with normal subjects due to the LCD. These data reinforce the possibility that Arg acts as a reservoir for increased ammonia detoxification instead of being used as a carbon skeleton donor.

Exercise has been proposed to have a biphasic effect on immune function [27], with various immune cell functions temporarily impaired following acute bouts of intense exercise [5]. In this study, we observed an increase in the number of leukocytes after exercise. We did not find changes in either packed cell volume, which is an internal control for volemic changes, or thrombocytes (data not shown). We did not detect a significant increase in the eosinophil or neutrophil count in response to either exercise or Arg supplementation. In contrast, we found a significant effect of supplementation on basophils and lymphocytes in response to exercise.

Distinct effects on white blood cells due to exercise have been reported in previous studies. In a study on heavy-resistance exercise, Kraemer et al. [28] reported a decrease in eosinophils, which was contradicted by later studies that showed an increase in the total leukocyte count without differences in specific leukocyte counts [29]. Even with an increase in the neutrophil count of 50–70% in some athletes, neutrophil levels did not change significantly in response to exercise in our study, which was expected based on previous reports [30]. Little is known about the response of granulocytes to acute exercise. However, some data have suggested that neutrophils increase following acute exercise, which is similar to the neutrophil increase caused by trauma [31], and that high-intensity exercise decreases neutrophil and thrombocyte adhesion [32]. These findings together can help explain our results.

An increase in leukocytes after acute exercise was extensively described in a review by Gleeson [5]. In our study, we found a 75–85% increase in leukocytes. This increase was mainly due to an increase in lymphocytes, which agreed with a previous report [30]. Interestingly, we also observed protection against the leukocyte and lymphocyte increases in the RG. Carbohydrate supplementation decreases both leukocyte and lymphocyte trafficking during exercise and attenuates lymphocytosis after acute exhaustive resistance [33]. Our data rule out a protective effect of Arg against the leukocytosis that might occur due to changes in glycemia.

A previous report by Sureda et al. [21] showed that neutrophilia and lymphopenia occurred after exhaustive exercise with constant plasma concentrations of Arg and ornithine but decreased citrulline. Supplementation with 3 g·day^-1^ Arg can increase the availability of Arg, ornithine and citrulline [18]. Because we used 100 mg·kg^-1^·day^-1^ (6.5–12.0 g·day^-1^), the supplementation used in our experiments may have resulted in an increased reservoir of these urea cycle intermediates [18].

A limitation of our study is the absence of blood amino acid measurements. Indeed, in another set of data, we measured blood amino acid levels after Arg supplementation, showing that this time frame was sufficient for Arg absorption (unpublished data). In this study, we showed a high correlation between the increases in the lymphocyte count and blood ammonia, both of which were prevented by Arg supplementation. In an elegant study, Garg et al. [34] recently proposed that T cells could act in concert with glia to protect neurons. This protection occurs via the liberation of lactate and glutamate from T cells following the release of cysteine (a precursor of glutathione synthesis) by astrocytes to protect neurons and the release of lactate to feed the neurons. Previous reports have also shown metabolic protection from lymphocytes in target tissues, including the maintenance of cognition [35-37]. In addition, our data show that the increase in blood globulins is affected by Arg supplementation. Given these data, we propose that increases in serum lymphocytes could be related to changes in ammonemia and ammonia metabolism.

## Conclusions

The modulation of arginine through supplementation in exercise is well established. In this study, we induced transitory hyperammonemia with a low carbohydrate diet and high intensity exercise to evaluate the changes in nitrogen metabolism.

Even with a six-fold increase in ammonemia during our protocol, we did not demonstrate either acute muscle damage or changes in glycemia. These data suggest that exercise is an efficient model to apply in sports medicine and nutrition.

Here, we showed for the first time that arginine supplementation decreases both ammonemia and the lymphocyte response during intense exercise and that the use of this amino acid can be a strategy to modify metabolism during exercise.

## Competing interests

The authors declare that they have no competing interests.

## Financial competing interests

The authors declare that they have no financial competing interests.

## Authors’ contributions

LCG, ABessa, RFD, ABassini and LCC: essential contributions to the conception and design of the study protocol; acquisition, analysis and interpretation of data; and involvement in drafting of the manuscript. RL, JPSWC, ABassini and LCC: critical revisions for important intellectual content. All authors read and approved the final manuscript.
